# Supplementary material for: Xanthine Oxidase Inhibitory Peptides from Larimichthys polyactis: Characterization and In Vitro/In Silico Evidence
Source: Foods. 2023 Feb 25;12(5):982. doi: 10.3390/foods12050982 (PMC10001067; doi:10.3390/foods12050982)
Supplement: Supplementary file 1 [file foods-12-00982-s001.zip › foods-2164179-supplementary.pdf]

foods
MDPI

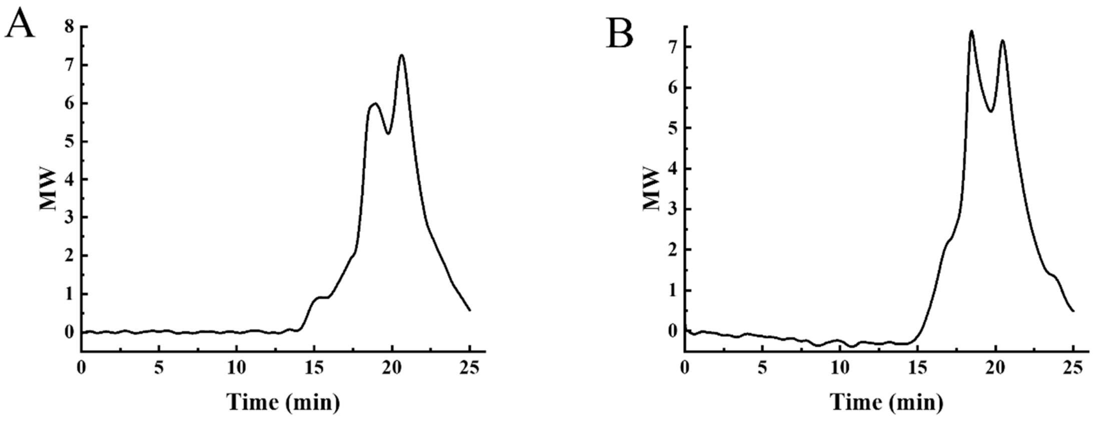

Supplementary Figure S1. MW distributions of small yellow croaker hydrolysates (A) and peptides molecular weight less than 3 kDa (B).

Supplementary Table S1. Relative peak table of small yellow croaker hydrolysates MW distributions.

Distribution	Mn (Daltons)	Mw (Daltons)	MP (Daltons)	Mz (Daltons)	Mz+1 (Daltons)	Slice Area
1	14,787	17,973	13,587	22,127	26,694	583
2	2744	3381	1558	4219	5073	1335
3	591	686	583	804	929	5947

Supplementary Table S2. Relative peak table of peptides molecular weight less than 3 kDa MW distributions.

Distribution	Mn (Daltons)	Mw (Daltons)	MP (Daltons)	Mz (Daltons)	Mz+1 (Daltons)	Slice Area
1	3884	511	2197	7461	11,014	1694
2	672	827	800	1039	1277	7577
